# Circulating microRNA Profiles during the Bovine Oestrous Cycle

**DOI:** 10.1371/journal.pone.0158160

**Published:** 2016-06-24

**Authors:** Jason Ioannidis, F. Xavier Donadeu

**Affiliations:** The Roslin Institute and R(D)SVS, University of Edinburgh, Easter Bush, Midlothian, United Kingdom; IPMC, CNRS UMR 7275 UNS, FRANCE

## Abstract

Up to 50% of ovulations go undetected in modern dairy herds due to attenuated oestrus behavior and a lack of high-accuracy methods for detection of fertile oestrus. This significantly reduces overall herd productivity and constitutes a high economic burden to the dairy industry. MicroRNAs (miRNAs) are ubiquitous regulators of gene expression during both health and disease and they have been shown to regulate different reproductive processes. Extracellular miRNAs are stable and can provide useful biomarkers of tissue function; changes in circulating miRNA profiles have been reported during menstrual cycles. This study sought to establish the potential of circulating miRNAs as biomarkers of oestrus in cattle. We collected plasma samples from 8 Holstein-Friesian heifers on days Days 0, 8 and 16 of an oestrous cycle and analysed small RNA populations on each Day using two independent high-throughput approaches, namely, Illumina sequencing (n = 24 samples) and Qiagen PCR arrays (n = 9 sample pools, 3–4 samples / pool). Subsequently, we used RT-qPCR (n = 24 samples) to validate the results of high-throughput analyses, as well as to establish the expression profiles of additional miRNAs previously reported to be differentially expressed during reproductive cycles. Overall, we identified four miRNAs (let-7f, miR-125b, miR-145 and miR-99a-5p), the plasma levels of which distinctly increased (up to 2.2-fold, P < 0.05) during oestrus (Day 0) relative to other stages of the cycle (Days 8 and 16). Moreover, we identified several hundred different isomiRs and established their relative abundance in bovine plasma. In summary, our results reveal the dynamic nature of plasma miRNAs during the oestrous cycle and provide evidence of the feasibility of using circulating miRNAs as biomarkers of reproductive function in livestock in the future.

## Introduction

Failure to adequately identify fertile oestrus in farmed animals is a major contributor to the long-standing problem of low fertility rates in modern production animal systems, particularly in cattle. To illustrate the severity of the problem, current conception rates at first postpartum service in dairy cows are approximately 40%; according to the UK’s Dairy Science Forum (2008), this is estimated to cost the UK dairy industry in excess of £300 million per year through extended calving intervals, in addition to being a major welfare issue through disease and premature culling.

The physiological period of anoestrus following calving is characterised by progressive restoration of the neuroendocrine-reproductive axis to allow adequate maturation of ovarian follicles followed by ovulation before normal oestrous cycles can be re-established [1]. In non-stressed dairy cows ovulatory activity normally begins within 2 to 3 weeks after calving. However, in modern dairy herds, a combination of factors resulting from both genetic selection for production traits and intensive management, including negative energy balance, stress and disease (uterine infection, mastitis, lameness), often disrupt normal neuroendocrine restoration which prevents normal follicle maturation and leads to a delay in the onset of regular, normal-length oestrous cycles [2, 3]. Furthermore, behavioural oestrus, physiologically triggered by high oestradiol levels from mature pre-ovulatory follicles, is significantly reduced under these conditions, both in intensity and duration; this leads to up to 50% of heats being missed by farm personnel [4]. Altogether, this leads to a dramatic decrease in breeding efficiency, increased risk of disease through non-productive inseminations, and extended calving intervals.

Periodic visual inspection of cows for behavioural signs of oestrus has been traditionally used to select cows for breeding [5]. More sophisticated methods include measurement of milk progesterone, heat mount detectors and activity monitors; in practice these are frequently combined with visual observation [5]. Yet, multiple reports agree that approximately 30% of oestrus events may still not be detected even when combinations of these methods are used [5, 6]. Thus, there is growing interest in the development of novel oestrus detection methods to minimise economic losses and boost annual milk production.

MiRNAs are small RNA molecules that play key roles as gene expression regulators in animal tissues [7]. Intracellular miRNAs enter the extracellular space [8] in the form of stable complexes within protective exosomes [9] and / or associated with proteins such as HDL and Ago2 [10, 11]. Some miRNAs are tissue-specific and are present in the circulation at quantifiable levels (e.g. placental miRNAs) [12]. These findings have led to a plethora of studies into the biomarker potential of circulating miRNAs; it is now widely accepted that miRNAs can offer useful circulating biomarkers of multiple diseases such as cancer, heart disease, autoimmune diseases [13, 14] as well as preeclampsia and other reproductive diseases [15, 16]. In contrast to the abundant literature focusing on humans, the potential of circulating miRNAs as biomarkers of animal disease and / or productivity traits has not been investigated in depth.

MiRNAs are involved in the regulation of follicular and luteal development (reviewed here [17]) as well as endometrial function [18]. Consistent with this, studies using cows and other species have identified numerous miRNAs with expression that changes significantly in association with follicle growth and selection, the follicle-to-luteal transition, and embryo implantation [19–21]. Because the reproductive tract is highly vascularised, particularly the ovary, it is reasonable to hypothesise that changes in miRNA levels occurring in those tissues may lead to changes in the levels of circulating miRNAs, which may provide useful oestrous cycle biomarkers. In this study we used next-generation sequencing and PCR-based platforms to compare miRNA profiles in the circulation of heifers across different stages of a normal oestrous cycle. We identified four circulating miRNAs, the levels of which were significantly higher during oestrus compared to other stages of the oestrous cycle. These findings pave the way towards the development of novel oestrus detection methods.

## Materials and Methods

### Experimental animals

Eight Holstein-Friesian heifers (15–17 months old) were oestrus-synchronised using Eazi-Breed^™^ CIDR^®^ Cattle Insert (1.38 g progesterone over eight days; Zoetis, USA), Receptal^®^ (0.02 mg buserelin on the day of CIDR insertion; MSD Animal Health, UK) and Estrumate^®^ (0.5 mg cloprostenol seven days after CIDR insertion; MSD Animal Health). Blood was collected on Days 0, 8 and 16 from all animals (Day 0 being the first day of observed oestrus). The stage of the oestrous cycle was confirmed by plasma progesterone profiles determined using a Coat-a-Count radio-immuno-assay (Siemens Healthcare, Germany). All animal procedures were carried out under the UK Home Office Animals (Scientific Procedures) Act 1986, license 60/4604, and with approval by the Ethical Review Committee, University of Edinburgh and all efforts were made to minimize animal suffering.

### Blood sample collection and processing

Blood was collected in 10 mL K2 EDTA Vacutainer tubes (Becton Dickinson, USA) by jugular venepuncture, using 18G needles (Becton Dickinson) and stored at 4°C. Within two hours of collection samples were centrifuged at 1,900 g for 10 min at 4°C to remove blood cells, and then again at 16,000 g for 10 min at 4°C to remove cellular debris and platelets. The second centrifugation step has been shown to significantly reduce platelet numbers in plasma samples, minimising platelet contamination [22]. In addition, haemolysis was controlled for by using absorbance at 414 nm and the ‘miR ratio’ (ΔCq between miR-451 and miR-23a) as described previously [23, 24]. All plasma samples were immediately frozen at -80°C.

### RNA extraction

RNA was extracted from 1.05 mL of plasma using TRIzol LS (Life Technologies, USA), following the manufacturer’s protocol. During the RNA extraction protocol, glycogen (180 μg; Sigma-Aldrich, USA) was added to each sample to facilitate the precipitation of RNA, and an exogenous miRNA control, syn-cel-miR-39-3p (0.25 fmol; Qiagen, NL), was spiked into each sample. RNA was re-suspended in 30 μL of RNase-free water and used immediately or frozen at -80°C.

### Small-RNA sequencing analysis

Small-RNA libraries were prepared using the Illumina TruSeq small-RNA sample preparation kit (Illumina, USA) following the manufacturer’s protocol. Libraries were submitted to 36-base single-end sequencing using the Illumina HiSeq 2000 platform. The raw sequencing data are available on the GEO database under accession GSE81050. Raw reads were processed using sRNAtoolbox 1.0; initially reads with no adaptor and/or with undetermined bases (N) were removed [25]. The bovine genome (assembly UMD 3.1, [26]) was used as reference; trimmed and quality-controlled reads were mapped against mature miRNAs from bovine and human (for homologue identification) in miRBase 20 (accessed 11/06/2014, [27]) allowing only a one-nucleotide mismatch; novel bovine miRNAs as well as isomiRs were identified at this stage.

Prediction of novel miRNAs was carried out using sRNAbench software, as described previously [28, 25]. Briefly, sequencing reads that did not map to known miRNAs were mapped to the bovine genome. Where pairs of read clusters mapped 60 nt apart, the two clusters (putative novel miRNAs) and the connecting sequence were tested for their ability to form a stem-loop (pre-miRNA). We also identified isomiRs of known canonical miRNAs using sRNAbench. Briefly, the procedure maps small RNA reads to pre-miRNA loop sequences, identifying differences in sequence using a step-wise approach (end-variations first, miRNA-body variations next, etc; for further details please refer to the sRNAbench manual). To increase confidence, only sequences present at a minimum of 25 RPM in more than 75% of samples analysed were taken as true isomiRs.

After mapping, human and bovine miRNA read counts were merged and normalised to generate reads per million mapped (RPMM). MiRNAs with less than 25 RPMMs in more than 75% of the samples within each of the experimental groups were excluded, keeping 181 miRNAs for further analysis. Normalised expression levels (RPMMs) from sRNAbench 1.0 were log2 transformed before applying repeated-measures ANOVA using “ez” package 4.2 [29], followed by FDR adjustment using R language 3.02 and RStudio 0.98 [30, 31]. The transformed data were normally distributed as determined by the D’Agostino-Pearson omnibus and Shapiro-Wilk normality tests. Statistical significance was set to FDR < 0.1. Sequencing data are provided in S1 File.

### PCR array analysis

To design our Custom PCR array we took all miRNAs included in the Qiagen Human miRNome^™^ miscript v. 16.0 PCR array and aligned them (using BLAST, [32]) to cow miRNA sequences listed in miRBase 19 (accessed 20/02/2013, [33]) in a Linux environment. A total of 308 miRNAs conserved in cow (i.e., with ≤ 2 nucleotide mismatches between human and cow sequences) were included in the 384-well Custom miScript miRNA PCR array (384-well, Qiagen).

Three sample pools from each of Day 0, Day 8 and Day 16 (3–4 samples / pool) were analysed. cDNA (10 μL) was synthesised from 2 μL of RNA sample using miScript II RT kit (Qiagen) in a Whatman-Biometra Thermocycler (Biometra, USA). The arrays were setup according to the manufacturer’s instructions and were analysed on the LightCycler 480 System (Roche, Switzerland). Data analysis was performed using Microsoft Excel (Microsoft Corporation, USA) and R programming using R language 3.02 and RStudio 0.98 [30, 31]. Raw Cq data were initially filtered to remove wells with non-specific amplification as identified by melting-curve analysis. Cq values were normalised using the global mean expression, which was calculated from miRNAs which were detected in all of the sample pools. The statistical analysis of the transformed normalised data was performed as described for the sequencing data above. The PCR array dataset is provided in S2 File.

### RT-qPCR analysis

cDNA was generated as described above and diluted for use in 10 μL qPCR reactions using Qiagen SYBR Green kits in an Agilent Mx3005P qPCR system (Agilent Technologies, USA). Raw fluorescence data were processed using Agilent MxPro software. A fluorescence threshold of 0.1 was used to determine Cq values for all experiments. The amplification efficiency ranged between 88% and 109%, with R2 > 0.85. Data were processed using Microsoft Excel (Microsoft Corporation). Statistical analyses were performed in GraphPad Prism 6 (GraphPad Software, USA) using repeated-measures ANOVA followed by Dunnett’s tests or 2-sample t-tests if comparisons involved only two means. Statistical significance was set to P < 0.05. For selected circulating miRNAs, normalised expression levels were correlated with plasma progesterone levels using Spearman’s correlation (ρ) in GraphPad Prism 6 (GraphPad Software).

### miRNA target analysis

Target analysis was carried out using DIANA miRPath 3.0 [34]. Briefly, experimentally validated miRNA targets were identified by calling DIANA TarBase 7.0 through the miRPath 3.0 interface [35]. Targets which were common to two or more of the miRNAs considered were further selected for pathway prediction using miRPath 3.0 selecting the gene intersection analysis method in the tool’s interface. Enriched pathways from the KEGG database were exported from the tool along with FDR-corrected p-values. A significance threshold of P = 0.05 was used. The predicted pathways (KEGG) are provided in S5 File.

## Results and Discussion

### Small-RNA sequencing of bovine plasma during the oestrous cycle

**Fig 1:**
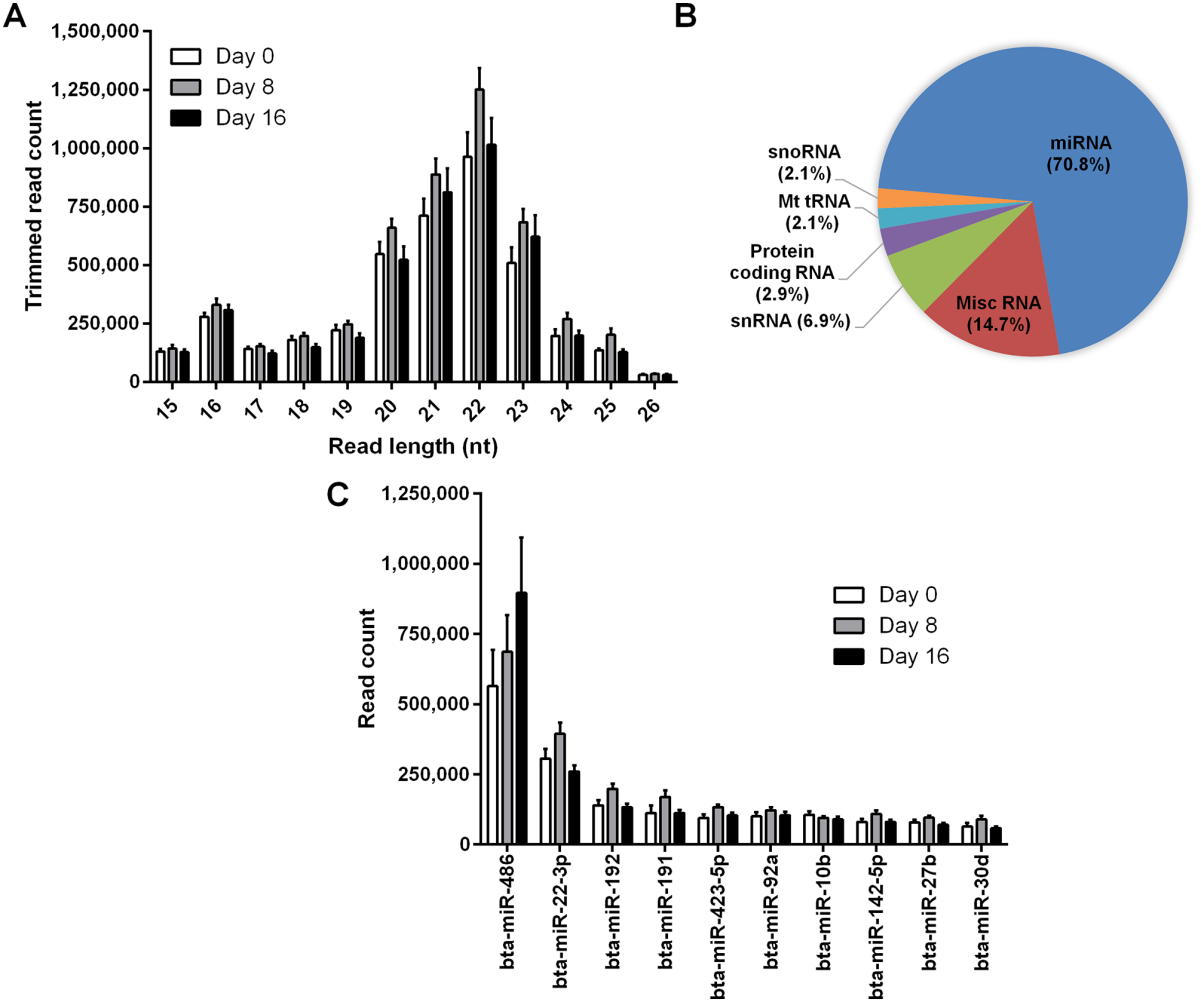
Results of small-RNA sequencing of bovine plasma. (A) Length distribution of trimmed read counts (mean number of reads ± SEM) for each of Days 0, 8 and 16 of the oestrous cycle (n = 8 heifers). (B) Relative abundance (% of total mapped reads) of different RNA species in bovine plasma. The ‘Misc RNA’ category predominantly includes Y RNA, Vault RNA and RNase P RNA. (C) Ten most abundant miRNAs (mean read counts ± SEM) in bovine plasma on each of Days 0, 8 and 16 of the oestrous cycle.

We sequenced 24 small-RNA libraries prepared from individual plasma samples collected from 8 non-pregnant heifers on each of Days 0, 8 and 16 of the oestrous cycle in order to identify differentially expressed miRNAs at oestrus (Day 0). On average, we obtained 9.1 million raw sequences from each sample (Table 1). The most common read lengths after removing the sequencing adaptors ranged between 20–23 nucleotides, which corresponds to the length of mature miRNAs (Fig 1A). On average, 4.3 million high quality reads (47.2% of raw reads) were mapped to the bovine genome per sample. More than 70% of these mapped reads corresponded to miRNAs, the majority of which were bovine (as registered in miRBase 20, accessed 11/06/2014 [36]); a much smaller fraction consisted of human miRNA homologues (0.1%) and predicted novel miRNAs (0.04%; Table 1). A small percentage of the mapped reads corresponded to other small non-coding structural and regulatory RNAs, such as small nuclear and nucleolar RNA (snRNA and snoRNA, respectively; Fig 1B).

**Table 1:** Summary showing various endpoints from small-RNA sequencing of plasma samples from Day 0, 8 and 16 of the oestrous cycle (n = 8 heifers).

	Day 0	Day 8	Day 16	Mean
**Raw reads**	9,241,040	9,380,798	8,737,868	9,119,902
**Reads with adapter**	8,461,507	8,361,521	7,918,324	8,247,117
**Reads that passed QC**	4,073,814	5,075,814	4,251,624	4,467,084
**Reads that mapped to the genome**	3,918,066	4,894,047	4,075,763	4,295,959
**Total miRNA reads**	2,727,909	3,468,900	2,922,572	3,039,794
**Bovine miRNA reads**	2,724,952	3,465,422	2,920,090	3,036,821
**Human miRNA reads**	2,957	3,479	2,482	2,973
**Novel miRNA reads**	1,493	2,034	1,597	1,700

On average, a total of 313 unique miRNAs (296 bovine and 17 human) were detected with more than 10 reads. Among the 10 most abundant miRNAs in plasma (Fig 1C), miR-486 (the most abundant) and miR-92a are reportedly expressed primarily in red blood cells [37], whereas miR-191 is highly expressed in platelets [38]. Four of the 10 most abundant miRNAs (miR-486, miR-92a, miR-192 and miR-423-5p) were also identified as highly abundant in bovine plasma in another study using Illumina technology [39]. The partial lack of agreement between the two studies could be explained by the use of different protocols to prepare the sequencing libraries as this has been shown to significantly influence the results of miRNA sequencing, mainly through effects on adaptor ligation [40].

**Fig 2:**
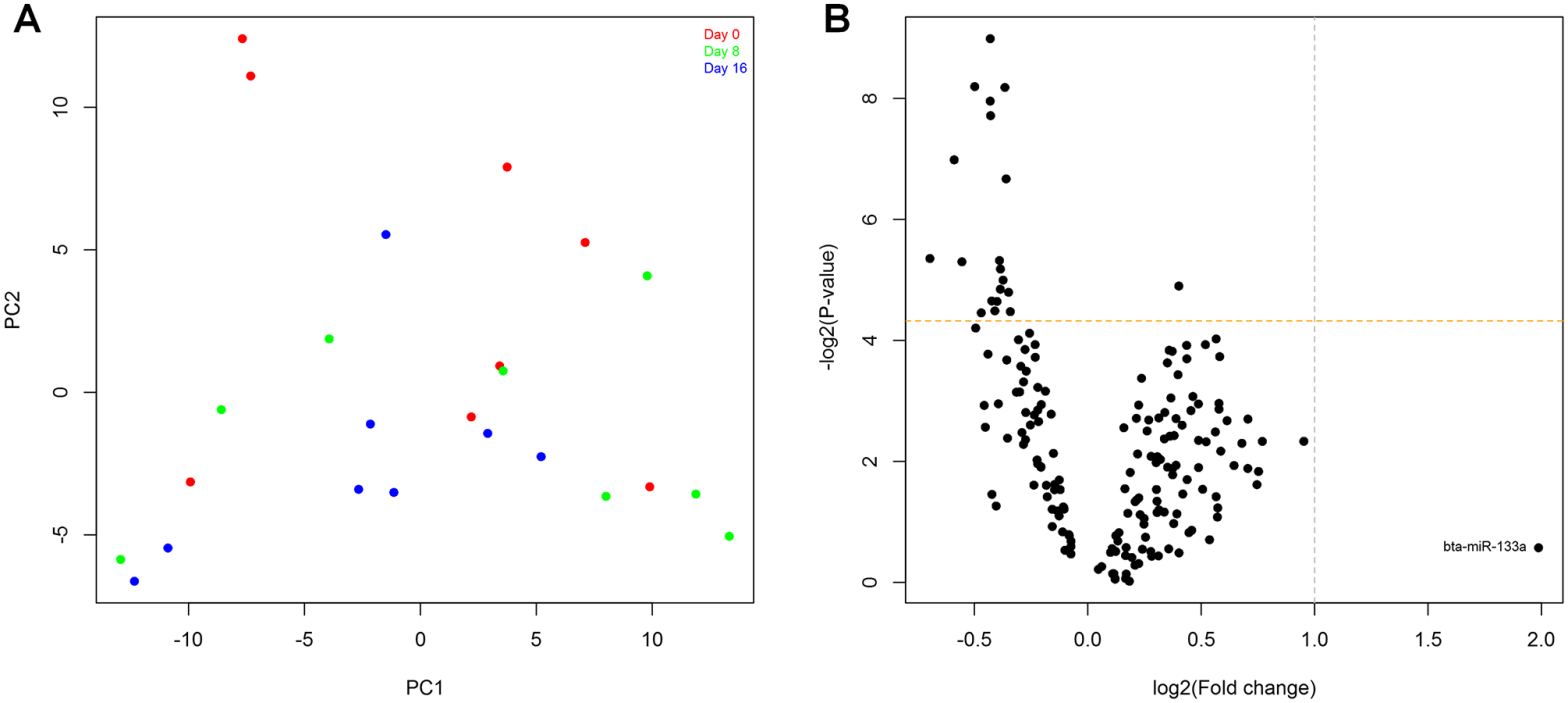
Differential expression analysis of small-RNA sequencing data from Days 0, 8 and 16 of the bovine oestrous cycle (n = 8 heifers). (A) PCA plot. (B) Volcano plot showing the largest fold-change between any 2 of the 3 days analysed for each miRNA. The grey dotted line indicates the 2-fold change threshold and the yellow horizontal line indicates P = 0.05. The plotted data have been log2 transformed.

Our differential expression analysis included 181 bovine and human miRNAs, all of which were present with more than 25 RPMM in at least 75% of samples in each experimental group (see Materials and Methods; S1 File). Principal component analysis did not reveal a clear separation of samples according to Day of the oestrous cycle (Fig 2A). Upon statistical analysis we detected an effect of Day of the oestrous cycle on the expression levels of 20 miRNAs (P < 0.05, Table 2; Fig 2B), however the differences involved were generally small (under 1.6-fold) and were not significant after multiple testing adjustment (FDR > 0.1), failing to identify a high-confidence candidate miRNA biomarker of oestrus at this stage of the study.

**Table 2:** Top miRNAs which were up- or down-regulated between different Days of the oestrous cycle using small RNA sequencing.

miRNA	D8/D0	D16/D0	D16/D8	P-value	FDR
**bta-miR-199a-5p**	0.72	0.62	0.99	0.024	0.405
**bta-miR-381**	0.71	0.66	1.06	0.008	0.238
bta-miR-125b	0.98	0.68	0.74	0.025	0.405
bta-miR-154c	0.71	0.71	1.07	0.003	0.172
bta-miR-100	0.88	0.72	0.91	0.046	0.405
bta-miR-214	0.78	0.74	1.01	0.002	0.172
bta-miR-199c	0.76	0.74	1.06	0.004	0.172
bta-miR-532	0.81	0.74	0.93	0.005	0.172
bta-miR-2285t	0.86	0.75	0.94	0.040	0.405
bta-miR-99b	0.90	0.75	0.90	0.045	0.405
bta-miR-380-3p	0.76	0.80	1.11	0.025	0.405
bta-miR-222	1.24	0.90	0.76	0.040	0.405
bta-miR-369-3p	0.77	0.79	1.11	0.028	0.405
bta-miR-10b	0.77	0.90	1.19	0.031	0.405
bta-miR-3431	0.79	0.77	1.06	0.035	0.405
bta-miR-224	0.84	0.78	0.97	0.003	0.172
bta-miR-23a	0.87	0.78	0.92	0.010	0.254
bta-miR-23b-3p	0.86	0.79	0.93	0.036	0.405
bta-miR-205	0.81	0.79	1.04	0.045	0.405
bta-miR-155	1.32	0.96	0.76	0.034	0.405

MiRNAs are sorted using the maximum fold-change across all three comparisons. MiRNAs with FC > 1.5 are in bold.

We also used our sequencing data to identify isomiRs in plasma samples using sRNAbench. We identified 655 different isomiRs for a total of 134 ‘canonical’ (miRBase) miRNAs. The majority of the isomiRs (507) involved modifications on the 3’ end of the ‘canonical’ miRNA, while others had modifications on the 5’ end (22 isomiRs) or the middle (13 isomiRs) of the ‘canonical’ miRNA. Ninety-three of the identified isomiRs contained multiple modifications (e.g. 5’ end addition and 3’ end addition). Finally, 20 isomiRs were multiple length variants involving a shift along the pre-miRNA sequence. A list of all the identified isomiRs is provided in S3 File. Interestingly, the canonical sequence was the most abundant in plasma for only 51 miRNAs (38.1%). For the remaining miRNAs, the most abundant sequence(s) corresponded to one or more of the identified isomiRs. For some miRNAs, the canonical sequence was expressed at much lower levels than the most abundant isomiR, this difference being ≥ 25-fold for a total of 21 miRNAs and up to 1044-fold for miR-192 (S3 File). This indicates that many bovine miRNA sequences listed on miRBase do not actually correspond to the most abundant isoform in plasma, and possibly also other tissues, something which will need to be considered in future studies. Because of the low abundance of most isomiRs, we did not use these data for differential expression analyses.

### PCR array profiling of miRNAs in bovine plasma during the oestrous cycle

To complement our sequencing analyses, we used a commercial Custom PCR array platform to profile the expression of 308 bovine miRNA in the same plasma samples. It is usually not feasible to screen a large number of individual samples using PCR arrays, therefore we analysed 3 plasma sample pools (3–4 samples / pool) from each of Days 0, 8 and 16 of the oestrous cycle.

**Fig 3:**
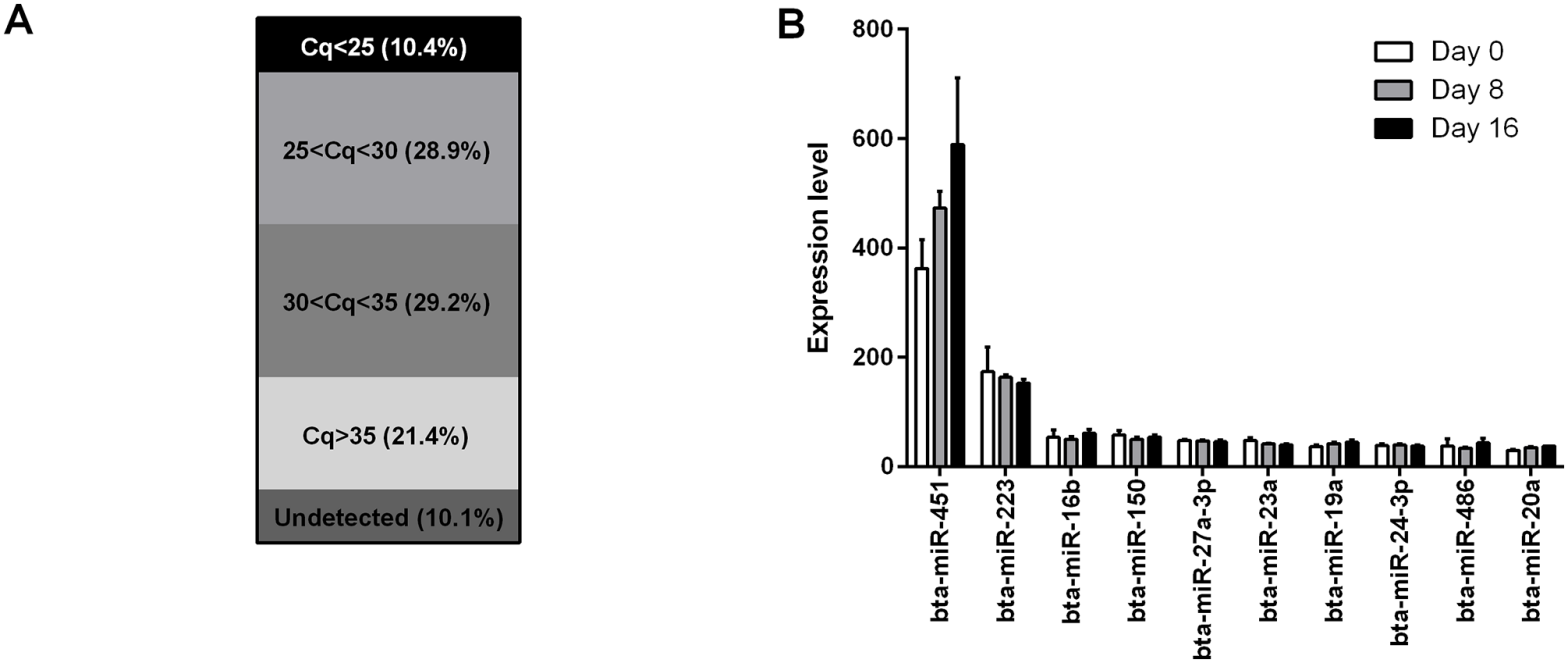
Results of PCR array analyses of bovine plasma miRNAs. (A) Distribution of C_q_ values. The percentage shown is the number of miRNAs in each category divided by the total number of miRNAs assayed. (B) The expression level (mean ± SEM of 2^^(40-Cq)^) of the top 10 miRNAs during each of Days 0, 8 and 16 of the oestrous cycle (n = 8 heifers).

A total of 211 miRNAs were detected at C_q_ < 35 across all samples (Fig 3A). The miRNAs which were most abundant in plasma (Fig 3B) in our experiment are reportedly expressed at high levels in blood cells, including erythrocytes (miR-451, miR-16b), leukocytes (miR-150, miR-27a, miR-23a) and thrombocytes (miR-223, miR-20a, miR-24) and are putatively released into the plasma through apoptosis, lysis or active shedding [41, 42, 14]. Out of the 20 most abundant miRNAs in each of the sequencing and PCR array datasets, only six (miR-451, miR-486, miR-22-3p, miR-92a, miR-191 and miR-140) were common to both datasets (20% overlap). A very abundant miRNA in the sequencing dataset (miR-21-5p) could not be compared as it was not included in the PCR array. However, considering the 150 most abundant miRNAs, 104 (70%) were detected by both platforms; out of the 46 miRNAs which were present only in the sequencing dataset, 35 were not represented on the PCR array, explaining a large part of the apparent lack of overlap in detected miRNAs between the two platforms. Further explanation for differences in the most abundant miRNAs is provided in the form of platform-specific biases involving, for example, sequencing adaptor ligation bias and differences in primer efficiency, which determine the relative abundance for a given miRNA [43, 44].

**Fig 4:**
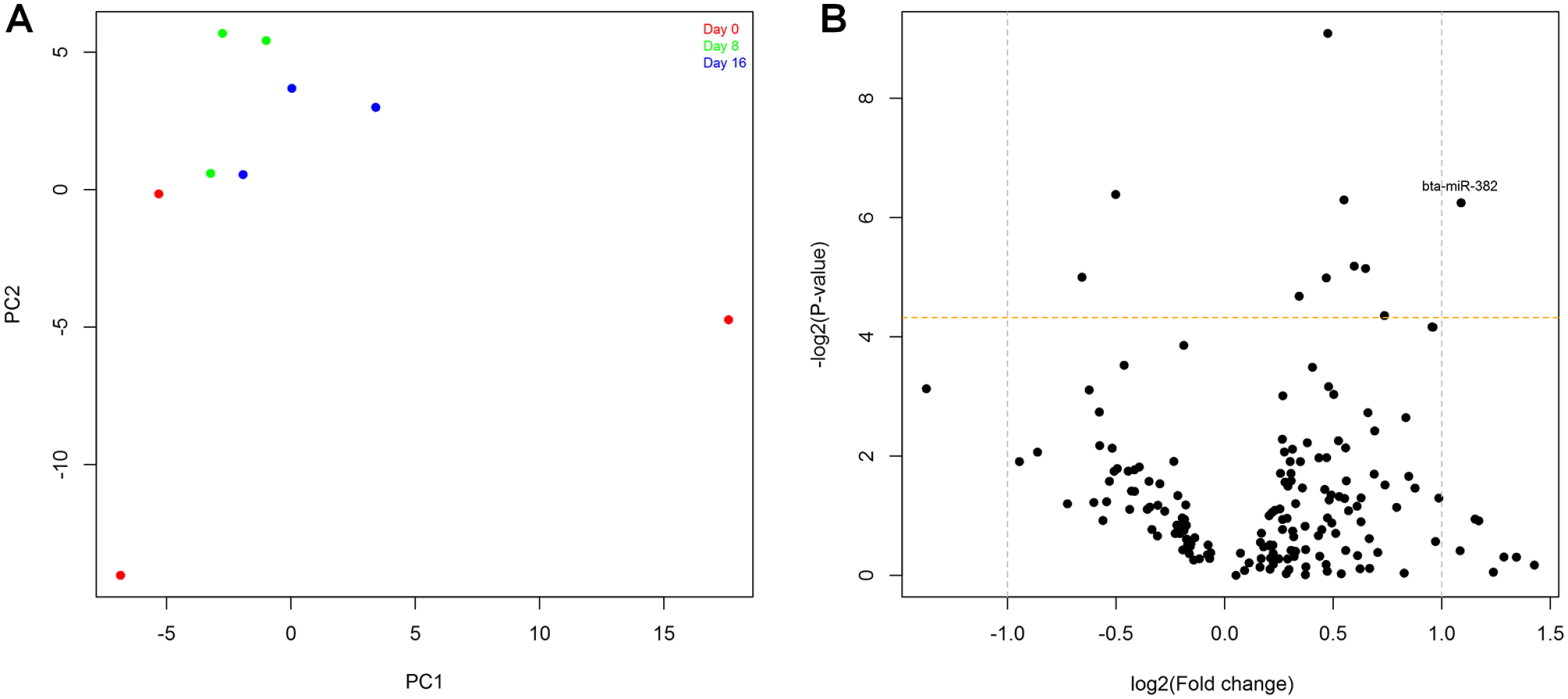
Differential expression analysis of PCR array data from Days 0, 8 and 16 of the bovine oestrous cycle (n = 8 heifers). (A) PCA plot. (B) Volcano plot showing the largest fold-change between any 2 of the 3 days analysed for each miRNA. The grey dotted lines indicate the 2-fold change threshold and the yellow lines indicate P = 0.05. The plotted data have been log2 transformed.

Principal component analysis did not reveal clear separation of the sample pools according to Day of the oestrous cycle (Fig 4A), although sample pools for Day 0 were much more spread than those for Days 8 and 16. After examining the major components in our PCA analysis we could not identify an obvious miRNA or biologically meaningful miRNA group which accounted for the variation within Day 0, although the low number of biological replicates used in the PCR array analyses may have been a contributing factor. A total of 169 miRNAs which had C_q_ < 35 in more than 67% of samples within each experimental group were used for statistical analyses (S2 File). Differences in expression across the oestrous cycle (fold-change ≤ 2.1) were identified for 10 miRNAs, although they did not reach significance after multiple testing adjustment (FDR > 0.1, Fig 4B, Table 3). Five of these miRNAs changed by more than 1.5-fold between Days (Fig 4B, Table 3) and only miR-224 was common to both PCR array and sequencing datasets (Tables 2 and 3).

**Table 3:** Top miRNAs which were up- or down-regulated between different Days of the oestrous cycle using PCR arrays.

miRNA	D8/D0	D16/D0	D16/D8	P-value	FDR
**bta-miR-224**	0.75	0.63	0.86	0.031	0.666
**bta-miR-185**	1.30	1.51	1.18	0.027	0.666
**bta-miR-140**	1.57	1.36	0.87	0.028	0.666
**bta-miR-455-5p**	1.67	1.61	0.99	0.049	0.787
**bta-miR-382**	0.48	1.00	2.13	0.013	0.556
bta-let-7f	0.71	0.87	1.26	0.012	0.556
bta-miR-20a	1.19	1.27	1.07	0.039	0.732
bta-miR-423-3p	1.38	1.23	0.89	0.032	0.666
bta-miR-106b	1.33	1.39	1.05	0.002	0.311
bta-miR-378	1.46	1.22	0.83	0.013	0.556

MiRNAs are sorted using the maximum fold-change across all three comparisons and miRNAs with FC > 1.5 are shown in bold.

### RT-qPCR validation of plasma miRNA profiles

Despite the lack of significant differences in miRNA levels after FDR adjustment in either of the sequencing and PCR array datasets, we decided to further analyse some of the differences identified with P < 0.05. For this, we performed RT-qPCR on individual plasma samples, which is considered the ‘gold standard’ for validation of high-throughput analyses results due to its relative high accuracy, the ability to factor-in the amplification efficiency for each miRNA and the absence of severe biases which can typically alter miRNA abundance data obtained from sequencing analyses [43, 45].

Using RT-qPCR we also profiled additional miRNAs which expression in body tissues changes during reproductive cycles according to previous reports [20, 21, 46–50]. This was adopted as a complementary approach to identify biologically relevant miRNAs which may have been missed by our high-throughput analyses.

**Fig 5:**
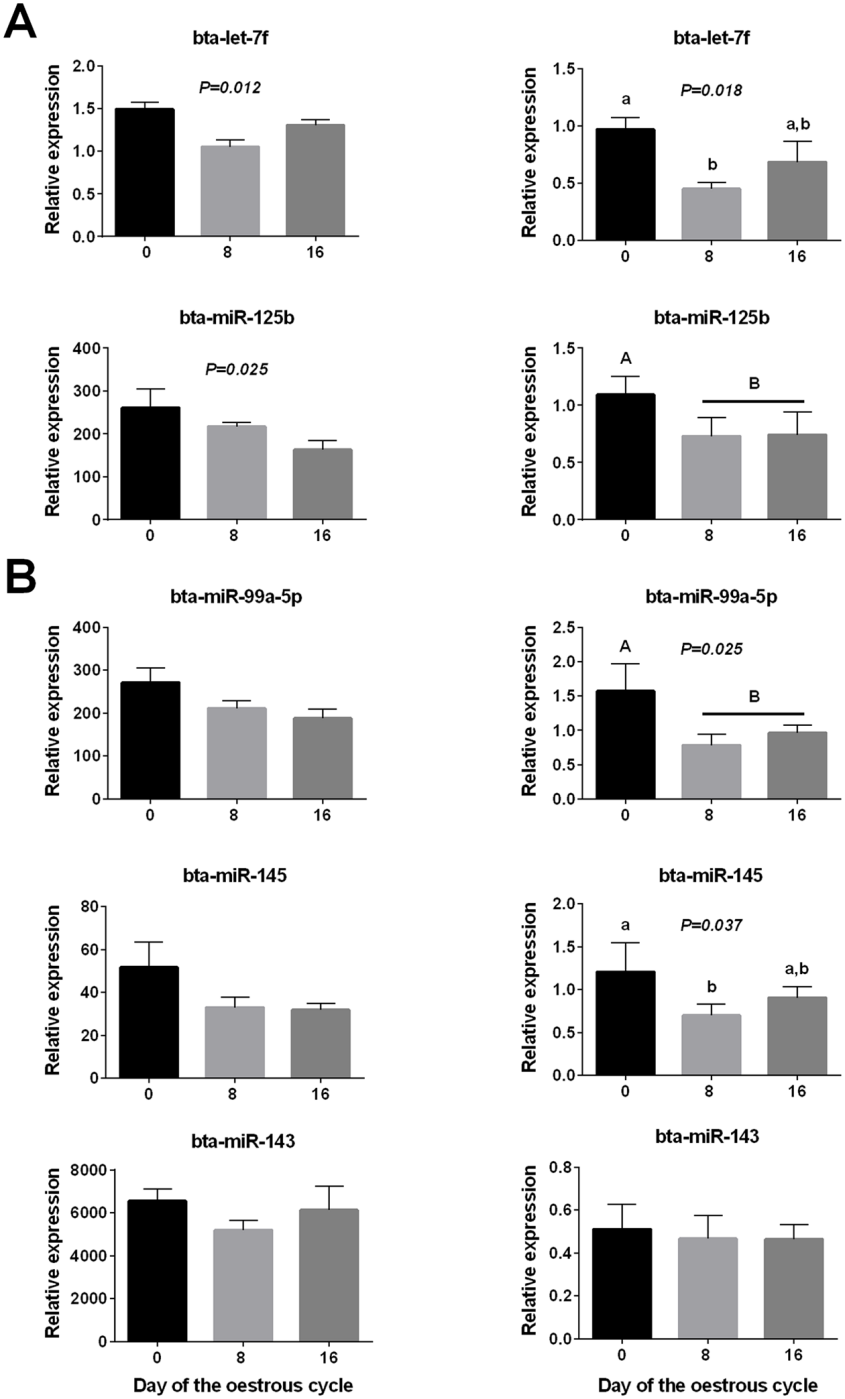
Results of RT-qPCR analyses of plasma miRNAs during the oestrous cycle (n = 8 heifers). (A) RT-qPCR validation (right column) of candidate plasma miRNAs that had been identified as differentially expressed by high-throughput analyses (small RNA sequencing or PCR array; shown in left column). (B) Expression profiles obtained by RT-qPCR (right column) of miRNAs previously reported to be differentially expressed in the ovary or endometrium during the oestrous cycle. Profiles for the same miRNAs obtained by small RNA sequencing are shown in the left column. In both (A) and (B) P-values from repeated-measures ANOVA are shown. Mean differences are indicated by different letters; a, b for P < 0.05 (Dunnett’s tests) and A, B for P < 0.1 (t-test).

We selected 8 miRNA candidates identified by sequencing (miR-125b, miR-155, miR-199a-5p, miR-381, miR-99b; Table 2) or PCR array (let-7f, miR-378, miR-455-5p; Table 3) for RT-qPCR analysis. Unfortunately, the plasma levels of miR-199a-5p, miR-381 and miR-99b were too low for accurate profiling by RT-qPCR (C_q_ > 35). Out of the remaining 5 miRNAs, miR-155, miR-378 and miR-455-5p did not change (P > 0.1) during the oestrous cycle when analysed by RT-qPCR. In contrast, let-7f levels decreased 2.2-fold (P < 0.05) on Day 8 compared to oestrus (Day 0) followed by a non-significant increase in mean levels on Day 16, in agreement with high-throughput data (Fig 5A). In addition, for miR-125b, although an overall effect of Day of oestrous cycle was not found, expression levels tended to be higher (P = 0.08) during oestrus (Day 0 vs Days 8 and 16 combined). Consistent with these findings, comparison of miRNA and progesterone levels during Days 0, 8 and 16 within animals (S4 File) revealed a negative correlation between let-7f and progesterone during the oestrus cycle (ρ = -0.523, P = 0.009).

Thus, overall, 2 out of 5 miRNAs identified by either sequencing or PCR array were validated successfully by RT-qPCR. A recent study involving all major vendors of miRNA profiling technologies [45] assessed different quantitative miRNA gene expression platforms (hybridization, sequencing and RT-qPCR) and found concordance in differentially expressed miRNAs to be surprisingly low between quantification platforms (54.6% on average). Based on this, the qPCR validation rates observed in our study were not lower than expected, especially when considering 1) the difficulty of profiling miRNAs in plasma and 2) the magnitude of the differences we attempted to validate.

Following this, we profiled additional miRNAs which we and others previously found to be differentially expressed in ovarian [21, 19, 46, 51] or endometrial [48–50] tissues across different stages of the reproductive cycle, but did not come up as significantly different between Days of the oestrus cycle in our high-throughput analyses. We selected 8 miRNAs that were reported to be upregulated (miR-145, miR-143, miR-99a-5p) or downregulated (miR-155 and miR-142-3p, miR-132, miR-378) in follicular relative to luteal tissues of ruminants [19, 21] or differentially expressed in endometrium during the human menstrual cycle (miR-31) [48–50]. These analyses revealed significant changes in the plasma levels of both miR-99a-5p and miR-145 during Days 8 and 16 (Fig 5B) of the oestrous cycle compared to Day 0, in agreement with differences reported between ovarian follicular and luteal tissues during oestrous cycles [21]. Accordingly, the circulating levels of these miRNAs were negatively correlated with plasma progesterone levels (ρ = -0.407; P = 0.049 and ρ = -0.404; P = 0.05 for miR-99a-5p and miR-145, respectively; S4 File), similar to let-7f above. Higher mean levels of those two miRNAs during oestrus (Day 0) were also apparent from high throughput data, but they were not significant (Fig 5B, left column). These results underline the advantages of combining different analytical approaches for identification of changes in circulating miRNA levels, particularly when such changes are expected to be modest.

Two previous studies analysed circulating miRNA profiles during human menstrual cycles; one reported no changes [52] and the other reported an increase in miR-31 levels during the secretory phase of the cycle [48]. Differences in reproductive physiology between species and in the analytical platforms that were used could very well account for the variable results between studies, especially if changes in miRNA levels in circulation across reproductive cycles are small. Another study recently reported differences in plasma miRNA levels (including miR-26b-4p, miR-125b and miR-99a-3p) between naturally cycling heifers and heifers treated with FSH to induce ovarian hyper-stimulation, but did not report differences in plasma miRNA expression during natural oestrous cycles [53].

The origin and function of the miRNAs identified as increasing in plasma during oestrus is not known. A reasonable assumption is that they may result, at least partially, from changes in miRNA expression in the ovary, particularly given that mature follicles and especially corpora lutea (CL) are highly vascularised structures that may contribute significantly to miRNA populations in the systemic circulation. In support of this, all 4 miRNAs that were expressed at higher levels on Day 0 than Day 8 of the oestrus cycle (let-7f, miR-125b, miR-99a-5p and miR-145) are also known to be expressed at higher levels in pre-ovulatory follicles than in corpora lutea of ruminant ovaries, consistent with their role in the follicle-to-luteal transition [21]. On the other hand, although some of the miRNAs analysed in this study are known to increase in expression in the ovary during the luteal phase (e.g. miR-132; [46, 54, 20]), they were not higher in plasma on Days 8 or 16 relative to Day 0 of the oestrus cycle. Moreover, the 4 miRNAs identified in our study to be differentially expressed during the oestrous cycle (let-7f, miR-125b, miR-99a-5p and miR-145; Fig 5) are indeed expressed naturally not only in the ovary but also (at lower levels) in many other tissues [55, 56], the relative contribution of which to circulating levels is not known. It is also worth pointing out that we have not been able to accurately quantify in plasma the gonad-specific miRNA, miR-202, despite this miRNA being present at relatively high levels in both follicular cells and follicular fluid [19, 51], a finding that, in the absence of any other evidence, argues against the ready transfer of at least some miRNAs from follicular tissues at levels that can be robustly quantified in circulation.

Finally, miRNA target analysis identified predicted KEGG pathways simultaneously targeted by two or more of the miRNAs identified in this study (miR-125b, let-7f, miR-99a-5p and miR-145), and those included ECM-receptor interaction, p53 signalling, Hippo signalling, Thyroid hormone and Cell cycle pathways (S5 File). However, the precise origin of these plasma miRNAs will need to be elucidated before meaningful conclusions about their role during the oestrus cycle can be drawn from these data.

In summary, the changes in miRNA levels during the bovine oestrous cycle identified in this study likely reflect changes in expression in tissues other than or in addition to the reproductive tract, a possibility that should be further investigated.

## Conclusions

Using a combination of small RNA sequencing and qPCR we have identified for the first time a subset of miRNAs with changing levels in plasma during the bovine oestrous cycle. Specifically, we identified an increase (up to 2.2-fold) in the levels of let-7f, miR-125b, miR-99a-5p and miR-145 during oestrus. These may reflect changes in miRNA expression in reproductive and/or other body tissues possibly involved in regulating cyclic reproductive activity. In addition, we have characterised and provide a list of miRNA isoforms together with their relative abundance in bovine plasma, which will provide valuable information for future studies. Our results pave the way towards exploring the role of circulating miRNAs as biomarkers of reproductive function in livestock. Future improvements in nucleic acid profiling technology may allow more accurate measurement of low-abundance miRNA levels in circulation and the identification of additional miRNA candidate biomarkers.

## Supporting Information

S1 File Small-RNA sequencing dataset. Read counts and differential expression analysis for bovine and human-homologue miRNAs in bovine plasma. FDR > 0.1 for all statistical results.
(XLSX)

S2 File PCR array dataset. C_q_ values and differential expression analysis results for bovine miRNAs in plasma measured by Qiagen PCR arrays. FDR > 0.1 for all statistical results.
(XLSX)

S3 File IsomiR dataset. A list of isomiRs in bovine plasma identified using sequencing.
(XLSX)

S4 File Plasma progesterone levels. Plasma progesterone levels on Days 0, 8 and 16 of the oestrous cycle, determined using a Coat-a-Count radio-immuno-assay.
(TIF)

S5 File Target and pathway analysis data. KEGG pathways enriched among experimentally validated targets of let-7f, miR-125b, miR-99a-5p and miR-145 using miRPath 3.0 and TarBase 7.0.
(XLSX)
