# Supplementary figures and images for: Circulating microRNA Profiles during the Bovine Oestrous Cycle
Source: PLoS One. 2016 Jun 24;11(6):e0158160. doi: 10.1371/journal.pone.0158160 (PMC4920432; doi:10.1371/journal.pone.0158160)

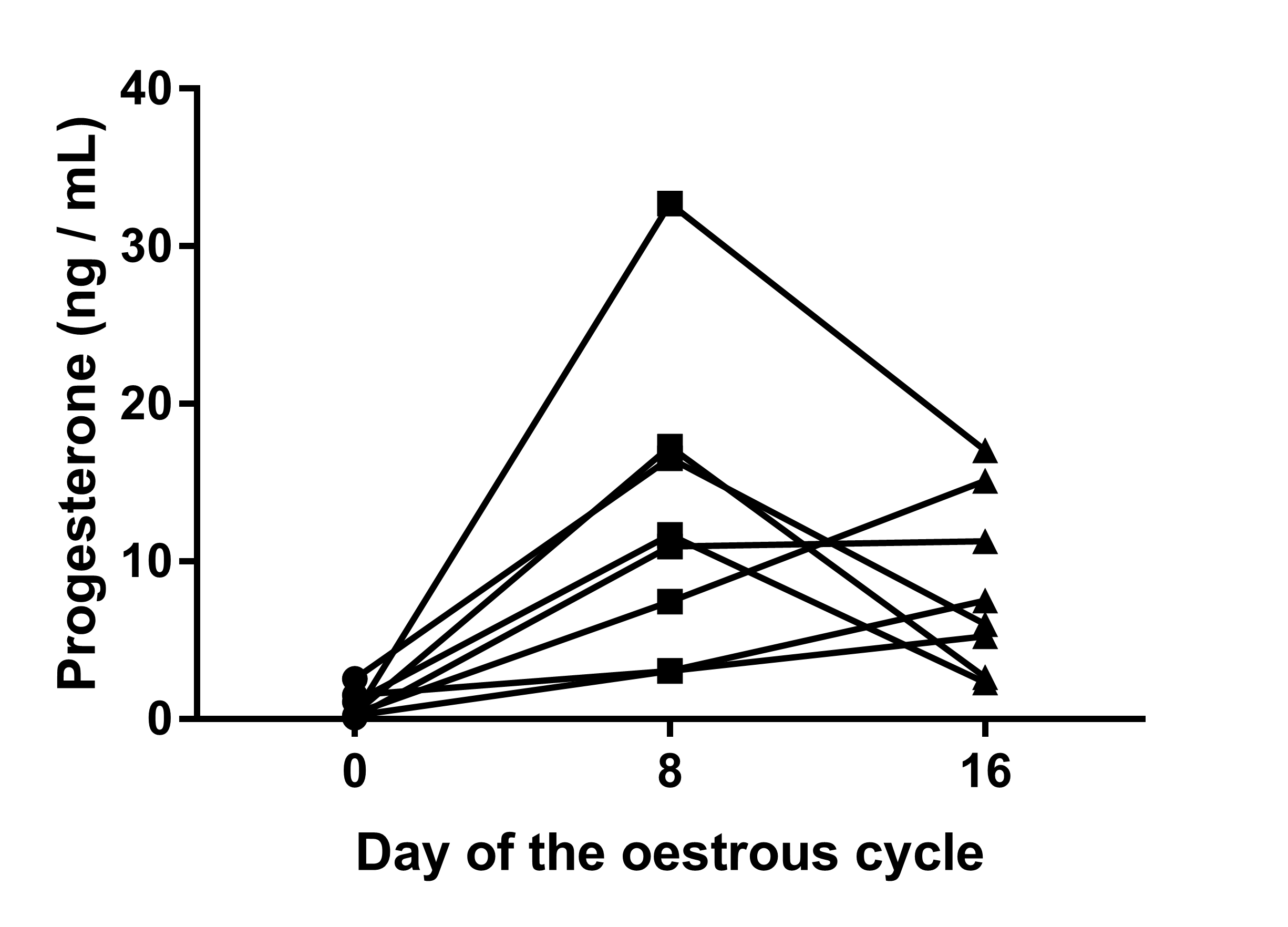

Supplement: S4 File — Plasma progesterone levels on Days 0, 8 and 16 of the oestrous cycle, determined using a Coat-a-Count radio-immuno-assay. (TIF) [file pone.0158160.s004.tif]
